# Mentoring SMART Women: Designing an Online Mentorship Training for Sheet Metal Workers to Increase Safety and Wellbeing

**DOI:** 10.1080/15578771.2026.2713214

**Published:** 2026-08-11

**Authors:** Lily M. Monsey, Hendrika W. Meischke, Marcy Miller, Noah S. Seixas, Emilia F. Vignola, Marissa G. Baker

**Affiliations:** a Department of Environmental and Occupational Health Sciences, University of Washington, Seattle, WA, USA; b Department of Health Services, University of Washington, Seattle, WA, USA; c Independent Contractor, M Miller LLC, Seattle, WA, USA; d Department of Health and Human Performance at York College, City University of New York, New York, NY, USA

**Keywords:** Tradeswomen, mentorship, training, construction education

## Abstract

Women make up just 4.3% of the national construction and extraction workforce in the USA as of 2025. These workers are exposed to unique physical and psychosocial hazards on the job. This article describes the development and formative testing of an online mentorship training program that equips union-affiliated, journey-level sheet metal workers with mentorship skills to support tradeswomen apprentice mentees in navigating a male-dominated work environment. The program included self-paced modules and live video conferencing sessions and was designed with input from allied partners in the construction industry. It was informed by key educational frameworks and health promotion theories and was pilot tested to a cohort of mentors. Thirty three journey-level mentors were invited to register for the online course. Thirty accessed the content and 28 completed a majority of the content. Of the 27 mentors who responded to the training evaluation survey, all agreed or strongly agreed that the training increased their confidence in mentoring, and all agreed or strongly agreed that the training advanced their understanding of mentoring relationships.

## Introduction

In the construction trades, recruiting and retaining tradeswomen is essential to advancing gender equity and economic mobility among women workers (Moir et al., 2011). As the industry continues to grow, there is an increasing need to recruit and retain a diverse workforce, including more women, to meet industry demands (Ariane Hegewisch, 2021). The number of women working in the construction trades increased from 3.9% to 4.3% between 2021 and 2025, signaling a positive shift in tradeswomen representation overall (US Bureau of Labor Statistics, 2022, 2026). Compared to their male counterparts, women workers continue to face heightened risks that make entering and remaining in the trades difficult, contributing to their minority status (CPWR Women in Construction [CPWR], 2025; Bridges et al., 2022; Goldenhar et al., 1998). To support tradeswomen participation, increased awareness and action around these heightened risks is essential. In addition to traditional physical hazards faced by all construction workers, tradeswomen are exposed to gendered hazards that have been associated with increased injury risk and stress (Curtis et al., 2018). These include hazing, harassment, overcompensation, ill-fitting PPE, lack of sanitary bathrooms, and lack of work-life balance (Curtis et al., 2021; Denissen & Saguy, 2014; Goldenhar & Sweeney, 1996; Turner et al., 2021). These risks contribute to the potential for further negative mental and physical health outcomes, as well as increased rates of attrition (Watts, 2009; Zhang et al., 2021). Below, we describe these gendered exposures in tradeswomen-focused literature in greater detail.

Gendered workplace hazards make it more difficult for women workers to enter and remain in the construction trades (Abid et al., 2021; Bridges et al., 2022; Curtis et al., 2021; Zhang et al., 2021). Ill-fitting PPE, such as gloves, boots, or harnesses that are too big, can make it difficult to perform work tasks safely (Curtis et al., 2016; Goldenhar & Sweeney, 1996). Tradeswomen report that they do not always have access to PPE that fits them properly (Curtis et al., 2021). Despite the need for appropriately sized equipment, some manufacturers do not make equipment in a wide enough range of sizes to fit all bodies (Denissen & Saguy, 2014). Tradeswomen also face specific gendered stereotypes from their male counterparts on the job, including assumptions that they are less competent than male workers, leading women to have to prove their abilities when their male counterparts would be assumed capable (Goldenhar & Sweeney, 1996; Holdsworth et al., 2023; Turner et al., 2021; Zhang et al., 2021). Women workers also face sexual harassment and discrimination on the job, which has been associated with higher perceived stress (Porter, 2019). Although some experienced women workers report that harassment is less prevalent than it was 30 years ago, tradeswomen still report that their male counterparts engage in various inappropriate behaviors on jobsites, including men posting nude photos of women in locker rooms, telling obscene jokes, and physically groping women (Curtis et al., 2021). The traditional norm of working overtime in the construction trades also creates barriers to success for tradeswomen with familial and other caretaking obligations (Ness, 2012). While tradeswomen report that greater representation of women in the field can help accommodate workers with familial obligations, these cultural and systemic challenges create persistent barriers to increased tradeswomen participation in the industry (Curtis et al., 2021; Ness, 2012; Zhang et al., 2021; Turner et al., 2021).

Despite these challenges, certain individual characteristics and workplace supports operate as empowering and protective factors. Women who feel fulfilled in their construction careers report heightened confidence and self-efficacy at work (Bridges et al., 2020). Research also highlights that women who report a sense of satisfaction in their careers can access a mindset in which they can actively reclaim what belonging and success in the trades can look like, enhancing their sense of wellbeing (Smith, 2013). In 2021, Turner et al. reported that resilience is considered a necessary attribute for women to thrive in the trades, despite little to no formal support from the workplace. Previous literature has highlighted tradeswomen strengths and organizational practices that support tradeswomen in navigating the complex social dynamics of the workplace (Bridges et al., 2022; Zhang et al., 2021). Support from instructors, supervisors, and allied coworkers is influential to success, with the presence of women in leadership as particularly meaningful (Bridges et al., 2020). Although literature reporting on mentorship for tradeswomen is scant, Menches and Abraham (2007) and Wright (2016) cite the broad value of mentoring networks and tradeswomen-supportive organizations as important to the retention of women workers. Tradeswomen-focused literature consistently highlights the need for policy, cultural, and organizational-level change to create an inclusive working environment for all workers (Bridges et al., 2022; Menches & Abraham, 2007; Smith, 2013; Turner et al., 2021).

While gender diversity initiatives in the construction trades are not new, industry compliance with the legislative and procedural requirements for gender quotas in the trades continues to fall short, signaling the need for increased support of women workers (Abid et al., 2021). Pre-apprenticeship programs, which are frequently administered by nonprofit organizations and are designed to provide potential new construction workers with key knowledge about the trades as well as physical and academic skills, present one strategy for recruiting more women into the industry (Wagner & Kulwiec, 2022). While these programs have been shown to help recruit more women and people of color, they typically last 10–20 weeks and are not typically designed to provide long-term support to incoming tradeswomen once they enter apprenticeship programs (Wagner & Kulwiec, 2022). In a small pilot mentorship program of tradeswomen in Washington state, the precursor to the trial in which this mentorship training was developed, tradeswomen focus group participants identified mentorship as a meaningful intervention to improve quality of life and mitigate the negative mental and physical impacts of harassment, aggression, and discrimination on the jobsite (Curtis et al., 2021). Support networks, including mentorship, have also been identified as an aproach to enable tradeswomen resilience and challenge dominant masculine culture by creating avenues for female leadership (Turner et al., 2021). Worksite mentorship can facilitate better safety communication practices and on-the-job training among workers (Kaskutas et al., 2013). To our knowledge, no research has reported on the steps involved in developing a tailored mentorship program for women in the sheet metal industry, nor on the efficacy of such training. Recent literature highlights how individual attributes function as mechanisms for success in an environment where systemic support that intentionally excludes women, signaling the need for interpersonal and organizational solutions (Bridges et al., 2020, 2022; Foley et al., 2022). Targeted mentorship programs that can be integrated into local contexts present a practical step to mitigate gendered exposures and improve workplace culture. The training presented here is a direct response to this tradeswomen-identified strategy to support safety, health, and wellbeing.

This mentorship training was developed as part of a Randomized Control Trial (RCT) with the Sheet Metal, Air, Rail, and Transportation (SMART) International Union. The goal of the program was to equip apprentice-level tradeswomen from SMART locals across the USA with the communication, goal setting, problem solving, and self-advocacy skills needed to navigate the sheet metal trade by training journey-level mentors through an online, self-paced training complemented with live online video conferencing group training sessions. The RCT was designed to test whether this intervention could improve safety, physical health, mental health, and retention for tradeswomen mentees. This manuscript provides an account of the development and formative testing of this mentorship training program with integrated reflections on how and why the training worked, supported by evaluation data.

## Literature review of educational and health promotion theories

The design and delivery of the mentorship training program was informed by one health promotion theory and two adult learning educational theories. The training was based on the idea that tradeswomen mentors are already experts in their fields with the knowledge they need to support apprentice mentees. The training harnessed specific social and communication skills to build confidence and self-efficacy through interactive skill practice and group-based discussion. Below, each theory is discussed in greater detail.

### Social cognitive theory

Social cognitive theory (SCT) was instrumental in the curricular design and facilitation of the program. Figure 1 shows an applied SCT model with mentoring as the target health behavior, with constructs tailored to the mentorship training context. COVID-19 influenced the delivery of the training, who could participate, and the conditions under which mentors engaged with mentees.

SCT brings together individual determinants with social structure to suggest that human agency and the social environment influence one another: environmental, cognitive, and behavioral factors function together to impact health behavior (Bandura, 2023; Bartholomew Eldredge et al., 2016). This model was selected because it acknowledges the environmental and contextual conditions that play a role in shaping mentor engagement. Cognitive factors such as *knowledge, expectations, values*, and *attitudes* are developed through training to shape mentoring behavior (Glanz et al., 2015). Behavioral factors such as *skills*, *practice*, and *self-efficacy* also influence mentoring behavior (Glanz et al., 2015; Strecher et al., 1986). Finally, environmental factors such as *social norms*, *influence on others*, *collective efficacy*, and *access in the community* that are specific to local-level culture and the culture among the mentor cohort come together to influence mentoring behavior (Glanz et al., 2015). In the facilitation of live online training sessions, educational techniques central to SCT, including *reinforcement, modeling*, and *tailoring*, and *role playing*, were used to build mentor confidence in a contained setting, allowing them to then engage more confidently with mentees (Bartholomew Eldredge et al., 2016; Strecher et al., 1986). Development of self*-efficacy* was central throughout the training process: *role modelling* and practicing *skills* in a group setting supported individual and collective confidence to engage in mentoring work. With this confidence, mentors could facilitate trusting relationships with their mentees, providing social support. In the long-term, sustained mentoring culture could lead to improve workplace culture and wellbeing (Curtis et al., 2021; Moir et al., 2011; Zhang et al., 2021). SCT has been used to guide previous health promotion interventions, including group-based and peer support programs focused on motivating nutritional change, physical movement, and knowledge around healthy lifestyle choices to support positive health outcomes (Islam et al., 2023; Whitehead, 2001). As constructs, *self-efficacy* and *observational learning* have been used frequently to motivate behavior change and model desired behavior from trusted sources (Islam et al., 2023).

### Andragogy

Malcolm Knowles’ concept of andragogy informed the design of the training curriculum. Andragogy is “the art and science of helping adults learn” and highlights the idea that adults absorb new knowledge best when they know why they are learning specific content, learn through hands-on practice, and can apply content in real-world situations (Knowles, 1984). To this end, each section of the online, self-paced training featured introductory videos focusing on why the topic was being explored and concluding sections with opportunities to apply the skill to a real-life scenario for working tradeswomen. In live online training sessions, discussion among the trained facilitator, senior mentor (an experienced tradeswoman who was able to provide more real-world ideas and reflections to the cohort compared to our study team, none of whom have lived experience working in the trades) and mentors focused on how the skills covered in the self-paced training could be used in real-life contexts; examples from mentors’ lived experience were highlighted in open discussion of how to integrate mentoring skills into everyday work. Previous research has highlighted the use of andragogic principles in a variety of health promotion and health contexts: it has been used to help individuals adhere to treatment plans, and self-manage behaviors associated with improved physical health (Niksadat et al., 2023). In educational spaces, using andragogic principles has been associated with students developing a deeper understanding of content and increasing student motivation to learn and participate in group settings (Krishna Nath Pandey, 2023).

### Bloom’s taxonomy

From educational theory, Bloom’s Taxonomy provided a framework for classifying learning objectives from the training into the hierarchy of human cognition; we used this framework to scaffold the mentor learning process through the self-paced and live portions of the online training (Mcdaniel, 2010). Bloom’s taxonomy delineates the process of *remembering, understanding, applying, analyzing, evaluating and creating* to span the process of building skill recognition of new concepts to using skills in real-life environments (Agarwal, 2019). These steps were used to develop the sequence of training activities as we introduced concepts foundational to skill development, gave examples of what these skills could look like in practice, and provided opportunities to practice applying skills in role-play scenarios before using them with their mentees in real life (Agarwal, 2019; Mcdaniel, 2010). Bloom’s taxonomy has been used in numerous educational settings to support critical thinking and retention of concepts among learners; it has been linked to high student satisfaction with how material is taught and the degree to which it helped them learn (Callaghan-Koru & Aqil, 2022).

## Methods

### Development of the mentorship training program

The development of the mentorship training program took place between March 2020 and April 2021. Figure 2 highlights each step of the training development process.

The training curriculum was developed by a research team within the University of Washington School of Public Health, including two professors of occupational health and safety, one professor of health communication, a study coordinator, and an educational consultant. The study team consulted three existing mentorship programs and one safety leadership program in the development process: content on active listening, methods mentors can use to contact mentees, strategies for dealing with challenging situations on jobsites, and steps to employing safety leadership via self-advocacy were used to inform training content (CPWR Primary Training Materials [CPWR], 2017; PROGRAM - Mentorship Matters [PROGRAM], n.d.). Study team members identified learning objectives designed to address specific psychosocial stressors unique to tradeswomen, and designed modules with content adapted from previous construction safety and mentorship programs and drafted original content tailored to tradeswomen experiences in collaboration with two expert tradeswomen allies. To translate the training into an online platform, the study team partnered with the Northwest Center for Public Health Practice at the University of Washington to create guided online modules that integrated visual, text, audio and video elements of each section of the training. Given the timeframe within which this training was developed and pilot tested, all sections of the training were adapted with COVID-19 in mind. The training was made shorter overall to ensure it was a manageable length for an online delivery. Content focused on the history of mentorship in the trades that was originally drafted was removed. Geven the pandemic setting, remote communication strategies for mentoring relationships were highlighted. External advisory board members including two experienced tradeswomen leaders, two occupational health and safety tradeswomen researchers, one union leader from SMART International and three construction health and safety leaders tested sections of the training and provided feedback on survey and evaluation instruments to ensure alignment with industry best practices. The study team sought additional support from our two experienced tradeswomen advisory board members to ensure that the training content was culturally relevant and that real-world examples were accurate and relevant before the training was launched for the first time in June 2021.

### Training content

The content of the online self-paced training includes learning objectives, structured through four thematic modules containing a total of 8 skill-based sections. Each section contains a combination of introductory and content-based videos, interactive problem-based scenarios, example audio content between fictitious mentors and mentees, and visual guides to describe and clarify concepts. Figure 3 shows the breakdown of each module and section of the training. The introductory module, *Building Foundations*, contains one section: Diving into Mentorship. This section outlines the program rationale, program components, and benefits to mentors, and the motivation to create a mentorship program for tradeswomen. The second module, the *Mentor Toolbox*, contains sections two, three, and four: Relationship Building, Goal Setting, and Active Listening. These sections highlight how to build a foundation of trust with your mentee and establish relationship norms, how to actively listen to your mentee’s experience, and how to help your mentee set small, achievable goals. The third module, the *Mentee Toolbox*, contains sections five and six: Problem Solving and Self-Advocacy. Problem Solving highlights how to walk through a five-step problem solving process to support your mentee in addressing any challenge they may be facing, and Self-Advocacy outlines a three-step process for supporting mentees to self-advocate for themselves on the job. The final module, *Moving Forward*, contains sections seven and eight: Navigating Challenges and Pulling it all Together. These sections outline common challenges in mentoring relationships and how to proactively address them, and how to use a combination of mentoring skills in real-life scenarios. Figure 3 also depicts each of the four live sessions: the kickoff, mentor skills, mentee skills, and moving forward. We provide more detailed descriptions and examples from each self-paced training and live session in the *detailed breakdown* subsection below.

Primary learning objectives of the training are to: 1) identify the benefits and need for mentoring skills for women in the trades; 2) describe the role of a mentor and the significance of trust in mentee relationships; 3) explain the importance of goal practices for measuring mentee success; 4) define active listening and use it to support mentees; 5) describe problem-solving approaches using the ADAPT model (assess *attitude*, *define* the problem, assess *alternative* solutions, *prepare* solutions, and *try* out the plan – this process is described in greater detail in the detailed training breakdown below); 6) identify steps mentees can take to become better advocates for themselves in the workplace; 7) explain strategies that mentees can use to navigate typical workplace challenges; and 8) describe the importance of using a combination of skills and reflection in a mentoring relationship. Knowledge-based quizzes were deliberately excluded from the online training to reduce association with a traditional, didactic learning environment. The goal was to reinforce that mentors are already experts in their own field; the skills provided in the training complement their lived experience.

### Recruitment and compensation

Mentors were recruited from 10 locals across the USA and Canada. The Assistant to the General President for SMART International selected coordinators at each local who identified potential mentors. Eligibility criteria required mentors to be journey-level workers, able to take the time to participate in online training, and willing to support at least one tradeswomen mentee for up to 1 year. One exception to these inclusion criteria was made to accommodate a tradeswoman who was an apprentice in the sheet metal trade with 20 years of experience working in a different trade; given her lived experience in a similarly male-dominated environment, she and the study team decided jointly that she would be a better fit for a mentor role. While the study restricted *mentee* participation to female tradeswomen, *mentors* of any gender interested in supporting tradeswomen were invited to participate. Although the study team encouraged project coordinators to reach out to any eligible journey-level worker they believed would be a good fit, most prospective mentors who expressed interest in the program were women. As a thank you for taking the time to complete the online training, mentors were also provided with a $100 gift card at the end of the training process, in addition to a $20 gift card for baseline and end-of-year surveys, described below.

### Feasibility testing

The mentorship training was first disseminated between June and October 2021 to a group of 19 mentors from across the USA. The training was disseminated a second time to a group of eight new mentors from across the USA and Canada in February–April 2023. To complement the online, self-paced training described below, four live online training sessions were conducted with each of the two mentor cohorts via video conferencing technology, held at deliberate intervals throughout the training process to create an opportunity for mentors to build rapport as a cohort, share mentoring experiences, and practice skills from the self-paced training in a live setting. Each live session was staggered to occur shortly after mentors were asked to complete a self-paced section of the training, providing a platform to discuss each skillset, practice skills, ask questions, and engage in peer support. To accommodate mentors’ very busy schedules, three meeting times for each live online session were provided to maximize attendance. Integral to these sessions was the presence of our senior mentor, who was a member of our external advisory board and a former tradeswoman mentor herself. She attended all live online training sessions and offered her expertise on navigating social dynamics within the trades.

Shortly after the second live training session and after they were asked to complete half of the self-paced training, mentors were matched with mentees according to both mentor and mentee preferencing process conducted by the study team. The study coordinator sent each mentor a paper copy of the mentor handbook, which provided a summary of each section of the training, space to log meetings with mentees, and worksheets to use with mentees. Given the geographically diverse and remote nature of the program, every mentor filled out a biography template with questions about their interests and history, communication style, what experiences brought them to mentorship, any specific skills or certifications they had, and what days and times they were available to meet with a mentee. These were sent out to mentees, and mentees shared their top three mentors with the study coordinator. The University of Washington study coordinator matched mentors with up to three mentees from within or outside their local union. In the third live training session, the study team provided mentors with preparation for their first meetings with their mentees. This included guidance forthe first few meetings, including conversation starters and establishing communication norms. Mentors were asked to establish a weekly or biweekly meeting cadence with mentees for a one-year period.

Figure 4 shows the breakdown of all training and mentorship-related activity on a chronological, one-year timeline, including periodic check-ins with the study coordinator and survey time points. Each live training session held was scheduled for 90 min; each online module was estimated to take between 15 minutes and 1 hour, depending on the number of skills covered. For access to the full online training and supporting materials, see the training availability statement.

### The training: a detailed breakdown

Below, we describe each module in the self-paced training and each live training session in detail. For each section, we highlight constructs from SCT (environmental, cognitive, and behavioral factors) that were operationalized through the training curriculum.

### Live session 1: the kickoff

The live kickoff session provided an overarching introduction to the program. The session was led and facilitated by a trained facilitator and attended by the study coordinator, study principal investigator, mentor support person, and leadership staff from some participating locals who wished to attend. In the 90-min session, mentors were introduced to the study team, given the chance to introduce themselves to each other, and share their motivation for joining the program. Here, *knowledge* and *expectations* as constructs from the SCT were operationalized to give mentors an overarching view of what the training and program would entail, and what they could expect to get out of the experience. The study coordinator shared further information about the goals of the mentorship program, the research study and the time commitment required to participate. Next steps to formally enroll in the program were discussed. Offline, newly enrolled mentors were guided to complete modules 1 and 2 of the self-paced training before the next live training session.

### Self-paced module 1: building foundations

In module 1, mentors learn about the overarching structure of the program and why it was created to support tradeswomen in section 1: Diving into Mentorship. The introductory video describes what mentorship is, how it can take many different forms, and how the program is designed to provide social and logistical support to apprentice mentees. Here, in addition to the *knowledge* and *expectations* constructs applied in the kickoff above, the study team sought to establish a positive *attitude* and common *values* among mentors to lay the foundation for a productive learning experience. In discussing the potential impact of mentorship more broadly, *influence on others* is highlighted to set mentors up with an understanding of how their role can positively shape mentee experiences. Information about previous pilot research is described to contextualize the current research, and the study team’s role and goals are addressed. Text and video content describe why mentorship is important for tradeswomen now and the benefits mentors and mentees can derive from mentorship. Logistical information about what to expect in the rest of the training is also discussed. This module encourages mentors to reflect on their own experience in the trades and emphasizes how their experience and knowledge position them as experts to support mentees; the training is designed to bolster their skills and confidence, building *self-efficacy*. This module is estimated to take 15–20 min to complete.

### Self-paced module 2: mentor toolbox

Module 2 consists of sections 2, 3, and 4 of the training: Relationship Building, Goal Practices, and Active Listening. All sections include an introductory video from the trained facilitator highlighting a definition of the skill, how it shows up in day-to-day settings, and a roadmap for how the skill will be taught in the coming section. Here, *expectations* are operationalized to lay the groundwork for the section and give mentors a sense of what they would be learning. In section 2, Relationship Building, mentors are provided text and video content to learn about different mentorship styles. This includes where mentors lie on the spectrum of relationships (compared to friendships, familial relationships, or supervisory relationships) and how to build a foundation of trust and boundaries early on in a mentor/ mentee relationship by establishing communication norms tailored to each mentor-mentee pair. This section also focuses on what the week-to-week work of mentorship looks like: how to create a meeting schedule, communicate changes in availability, and strategize for connecting in remote settings. Section 3, Goal Practices, focuses on how to help your mentee set and work toward small, achievable goals, harnessing *skills* as a guiding construct from SCT. This section also discusses how goal setting can be a way to structure mentor/mentee check-ins when there isn’t a specific “problem” at hand a mentee may be encountering. Specifically, this section outlines a specific goal-setting process mentors can use. Steps of the process include: 1) identifying long-term goals, 2) assessing your mentee’s progress, 3) how to support your mentee in what practices they want to continue, stop, and start, and 4) reviewing the plan for implementing small goals. This framework encourages mentors to help mentees identify small, measurable behaviors they want to continue taking in service to their goal, behaviors they want to stop that are currently working against their progress toward their goal, and new behaviors or actions they want to begin incorporating into their lives to help work toward their current goal. This section includes an audio call with the fictitious mentor (Val) and fictitious mentee (Kendra), demonstrating goal setting in conversation. Section 4, Active Listening, walks through the three steps of connecting, clarifying, and validating. This section includes another audio call between Val and Kendra in which Val uses the three steps of active listening. In sections 3 and 4, *skills* were operationalized by providing a breakdown of how to approach and use each skill with a mentee. *Observational learning* was operationalized through scenarios demonstrating the use of each skill in a practical scenario to support learning. Reflection points ask mentors to consider their own experience with similar situations sought to support mentors in understanding how each skill could emerge in their own work with mentees. This module is estimated to take 45–60 min to complete.

### Live session 2: mentor skills

The second live session, which focused on contextualizing skills from Modules 1 and 2, was held approximately 1 month after the kickoff session. The trained facilitator led the session, while our senior mentor attended to provide targeted support on specific skills and answer mentors’ questions. The session began with an overview of the relationship-building, goal setting, and active listening sections; summaries of the skills were reviewed, citing interactive examples from the self-paced training sections. The facilitator provided time for questions about the content and welcomed feedback from mentors about how they have seen these skills come up in their own experiences. In doing so, the facilitators addressed *self-efficacy* and *influence on others* to support mentors in applying skills from the training and recognizing where mentoring skills already show up in interactions. Next, a video narrated by scholar and motivational speaker Brené Brown, describing how to practice empathy in daily interactions, was played to promote discussion about best practices for active listening. Mentors shared their reactions to the video, and the facilitator asked mentors to consider how empathy plays a role in active listening. As a final activity, *observational learning* and *influence on others* were operationalized in a conversation between the senior mentor and a volunteer mentor. They modeled a conversation for how to set small, achievable goals, using a real-life example from the mentor’s current work life that was discussed with the mentor offline in a planning session preceding the training session. The remaining mentors were sent to “breakout rooms” to *practice* walking through the steps of goal setting using examples from their own lives. Lastly, the facilitator reviewed recommendations for approaching the first few meetings with mentees, in preparation for being matched with mentees several weeks thereafter. The study team concluded by asking mentors to reach out if they had any concerns about beginning to work with their mentees. Mentors with concerns or questions were contacted in a follow-up email by the study coordinator for individualized support.

In discussion after the session, the skilled facilitator and study coordinator reflected that there was too much emphasis on skill practice in this session and not enough generative discussion of how these skills show up in day-to-day workplace interactions that would link the skills to mentors’ and mentees’ working lives. Having mentors participate in the demonstration of goal setting had been a highlight; this gave mentors the chance to take on a leadership role in the training and develop confidence in their skills to support self-efficacy in mentoring interaction moving forward.

### Self-paced module 3: mentee toolbox

Module 3 includes sections 5 and 6: Problem Solving and Self-Advocacy. These sections focus on skills mentors can learn not only for themselves, but ones they can help mentees build through interactions on a worksite. Section 5 features a five step process (ADAPT): 1) checking in on “attitude” or one’s emotional state when approaching an issue; 2) “defining” the specific problem at hand; 3) creating multiple or “alternative” solutions to approach the problem at hand; 4) “preparing” solutions, or reflecting on which solutions to try first and thinking through how to approach a conversation beforehand; and lastly, 5) “trying out” solutions – actually putting a solution into action. This section features a click-through scenario in which the fictitious mentee, Kendra, needs to switch her hours due to a family commitment, and how she employs the ADAPT problem-solving process to identify a solution. In this problem-based scenario, the learner navigates to each new step of the story by clicking on an arrow and choosing different options for moving forward in the process. At the end of the section, each step is broken down in more depth, and an alternative scenario is presented in which the learner could walk through the process if they had selected a different option for moving forward. This interactive exercise provides the learner with the opportunity to operationalize skills (personal factor) and practice by using the knowledge from the ADAPT model and applying it to their decision-making through the exercise. Section 6, Self-Advocacy, details a 3-step process for verbally advocating for oneself: 1) open with the goal, 2) reframe the message, and 3) wrap it up. This section starts and ends with two audio calls between Val (mentor) and Kendra (mentee). In the first call, Kendra is upset that her foreman has assigned her cleaning tasks on her jobsite while others get to learn new skills. Through the call with Val, she identifies that she wants to self-advocate to her foreman to make a change. With Val’s support, she first identifies a clear statement with her desired outcome to make early in the conversation with her foreman. Second, she reframes her message to support consensus-building and collaboration to find a mutually agreeable solution. Third, she ends with a statement to prompt her foreman to help her find a course of action to meet her stated goal. The audio call format provides the space to operationalize *role modelling* and *skill development* from SCT to facilitate the learning process. This module is estimated to take 45–60 min to complete.

### Live session 3: mentee skills

The third live training session focused on two goals: providing mentors with time to practice skills covered in module 3 and discussing how the first few meetings with their mentees had gone since being matched. Guided by the facilitator, the session began with a discussion of how the first contacts with mentees had gone, including questions and clarifications about the mentorship role. Here, development of *social norms* was facilitated to help the mentor cohort build capacity to lead initial conversations with mentees. In this session, mentors also provided each other with recommendations for how to engage with their mentees, depending on challenges or successes they were experiencing, thereby facilitating group support to perform mentorship through *collective efficacy*. In the second half of the session, the facilitator briefly summarized content from Problem Solving and Self-Advocacy sections. After the study coordinator and senior mentor modeled a problem-solving conversation, mentors were split into pairs to practice problem solving strategies they could apply with their mentees. Here, mentors practiced *skills* to build *self-efficacy* in interacting with their mentees.

After this live session, reflection among the mentor support person, skilled facilitator, and study coordinator included discussion about the challenge of having slightly different groups of mentors present for each session; given that multiple times were held for each session, no one group was the same from session 1 to session 2, making it more challenging to build rapport among each group. Discussion also included the desire to create even more space for mentor-driven discussion, rather than setting discussion topics ahead of time; this was integrated into the third live session, with the study team reaching out to all mentors beforehand to ask if there were specific topics they wanted to dedicate discussion time to.

### Self-paced module 4: moving forward

Module 4 includes sections 7 and 8 of the training: Navigating Challenges and Putting it all Together. Section 7 brings up common challenges faced in mentoring relationships, such as falling out of touch for longer periods or needing to re-set boundaries with a mentee, and strategies to address these challenges directly. Section 8 presents no new content but provides an opportunity for the learner to use a combination of mentoring skills covered throughout the training in a multi-part, interactive scenario between a mentor and mentee. At each step of the way, the learner can choose one of two to three options for how to proceed through a scenario with branching logic; by clicking through with one choice, they learn what happens in the interaction depending on their selection, with some choices leading to enhancement of the mentor/mentee relationship and other choices leading to communication breakdowns. Learners can move back and forth to see how selecting a different option changes the outcome of the scenario. This section was designed to support learners in operationalizing their knowledge and skill development through the training, applying it to a new scenario where they can practice bringing their new knowledge together in one exercise. This module was estimated to take 45–60 min to complete.

### Live session 4: moving forward

The fourth live training session focused on how to navigate common challenges in mentorship and build communication practices to sustain mentoring relationships. Here, environmental constructs from SCT, such as *influence on others* and *access in the community*, were used to better understand barriers to mentorship and identify potential solutions. Unlike previous live sessions, which focused more heavily on reviewing and practicing content from the self-paced modules, this session was guided by mentor needs: before the session, the study coordinator sent out a survey to assess what topics mentors would like to focus on, giving the study team time to craft a session tailored to mentor preferences. The session focused primarily on working with mentees who were uncommunicative; working through past challenges from mentors’ own experiences that inform their mentorship styles; and finding time to simply connect with one another as they navigated what it meant to rise into greater leadership.

Reflecting after the session, the skilled facilitator, mentor support person, and study coordinator all agreed that this session felt the most successful due to a combination of rapport built through previous sessions among mentors, as well as the strong balance of skill practice and discussion. The skilled facilitator noted that in having found this balance, the level of trust gained by mentors was reflected in their willingness to engage fully and vulnerably; this was shown in mentors’ openness to contribute their insights and personal experiences to the discussion.

## Results

### Participants and evaluation measures

The mentorship training was evaluated through formative assessments after each live online session and a summative post-training survey at the conclusion of all training activities. Each of the formative surveys and the summative survey contained both multiple choice and open-ended questions. Table 1 summarizes descriptive analysis of multiple-choice questions from all formative surveys and the summative post-training survey. Table 2 summarizes a content analysis of open-ended responses from all formative surveys and the summative survey. The total number of mentors who completed the training was 27 over a 3-year period, encompassing two rounds of mentor training in 2021 and 2023. Twenty-six mentors identified as female, while one mentor identified as male. Of those who completed the training, 10 mentors attended all live sessions, 11 attended three sessions, four attended two sessions, and two attended only one session. Over the course of the training period, six mentors dropped out of the training program.

### Live online session feedback: formative assessments

Immediately after the second, third, and fourth live sessions, mentors were asked to fill out a two-question online survey about their experience, which was used by the study team to inform the design of subsequent live training sessions and to determine if follow-up with individual mentors was needed. No evaluation was sent after the kickoff session due to the informational focus and lack of educational content covered. Table 1 summarizes multiple-choice answers to the question *How valuable was today’s session?* on a 5-point Likert scale (not at all valuable, a little valuable, somewhat valuable, quite valuable, and very valuable).) Ninety-four, eighty-six, and one hundred percent of mentors who responded rated sessions as “quite” or “very” valuable for sessions two, three, and four, respectively. Each formative survey also included an open-ended question asking mentors to provide suggestions for how to make the training session more worthwhile. Top suggestions for improvement were to make sessions longer (19%) and provide more facilitation in breakout rooms (14%), and create more time for breakout rooms (13%). More time for real-world examples was also discussed. Table 2 highlights illustrative quotes for each response category.

### Summative post-training evaluation

Table 1 shows the results of the multiple-choice questions included in the summative post-training survey. Overall, 89% of mentors reported that the training increased confidence “somewhat” or “very much” across all mentoring skills covered in the training. Mentors reported that “understanding the role of boundaries, trust and confidentiality in a mentor/mentee relationship” and “understanding the importance of goal setting for mentee growth” were skills in which their confidence changed the most, with 67% of mentors reporting that their confidence changed “very much” in these areas. At least 70% of mentors reported an intention to use communication skills, goal setting skills, problem solving skills and self-advocacy skills “very much.” When rating the value of different training elements, the self-paced training and live Zoom training session were reported as most helpful to the learning process, with 70% and 67% of mentors reporting each element as very helpful, respectively. Forty-eight percent of mentors rated the mentor handbook as helping them learn “very much.” Thirty-five percent and 30% of mentors stated that the mentor workbook and live training sessions were “a little” or “somewhat” helpful to the learning process, respectively.

Table 2 summarizes mentor feedback on two open-ended questions from the summative post-training evaluation. Questions were designed for mentors to expand on the positive aspects of their experience and to share constructive suggestions for improving the training. To summarize these responses, a content analysis was conducted to become familiarized with the data, create inductive categories that were reflective of frequently occurring responses, and report the findings (Vaismoradi et al., 2016). The two prompts were optional, and not all mentors who completed the survey opted to answer them. Of the 27 mentors who responded to the survey, 24 responded to the first question, and 10 responded to the second prompt: *Please provide any suggestions for how the overall training could be improved*. For the first question, half of respondents reported discussion and/or connection with other mentors and the study team as the most valuable aspect of the training, while one third reported that skill development or practice was most valuable, and 13% reported that the training in general was valuable to them. Of those who responded to the second question, half suggested that more training opportunities would have improved their experience in the training, 20% made suggestions pertaining to the engagement strategies used in the online and live training, and 20% suggested changes to the training content to improve the overall training experience. Table 2 highlights quotes that illustrate each emergent category.

### Highlights from mentor check-ins

In study check-ins with mentors throughout each year-long mentorship period following the training, the University of Washington study coordinator checked in quarterly with all mentors to assess progress with mentees and provide additional logistical support as needed. These check-ins provided insight into the mentors’ growth and experience working with mentees. Many reported that the training supported their own growth through interaction with other mentors. One mentor reported that it gave her the confidence to rise into a leadership position at her local. Another mentor highlighted that the active listening process taught in the training (connect, clarify, validate) was one she used regularly with her mentee. When describing the value of the training, she shared,

I think just practicing the listening and repeating what I heard her say just to build up the communication. I felt that that exercise was really good for me … I use that exercise a lot.

(Mentor #1, personal communication, August 23, 2024)

Three months after the training, a third mentor shared a reflection from the training that spoke to the generational differences among tradeswomen, and specifically what kind of behavior from others should be considered tolerable and what should prompt intervention. After getting to hear other mentors’ approaches to helping their mentees with harassment, she realized she shouldn’t coach her mentee to simply “put up” with the kind of treatment she had to endure as an apprentice but support her in addressing the issue directly with the foreman on her jobsite. These interactions suggest that mentorship creates the space for reflection, skill building, and career growth for mentors.

## Discussion

To our knowledge, this is the first report describing the development and dissemination of a mentorship training program for tradeswomen. The majority of mentors who participated in the program reported increased confidence in the skills learned through training and at least three quarters of all mentors reported intent to use communication, goal setting, problem solving, and self-advocacy skills with their mentees. Open-ended responses from the summative post-training survey reveal that skill development and practice was the most frequently cited valuable element of the training, with discussion and connection with other mentors and/or the study team also reported frequently. Despite the study team’s effort to minimize burden on an already busy population, mentors reported that more training opportunities and longer training sessions would have enhanced their training experience. This suggests that adding optional training sessions may provide a valuable source of peer support without requiring mentors to set aside more time from their busy schedules. When asked to rate the value of the different training elements, 70% of mentors reported the self-paced training to be most valuable, while 67% cited the live training sessions as very valuable, suggesting that both the live and self-paced elements of the training were important elements in mentors’ overall experience. Future online mentorship efforts should consider balancing the benefits of more structured skill development with discussion of how these skills can be applied in real-world situations.

Formative feedback from the live training sessions showed that a majority of respondents found all sessions very valuable, with key feedback highlighting the need for more discussion, longer sessions, and greater facilitation in the breakout rooms. Mentors highlighted the value of hearing other women’s stories. This underscores the importance of developing group-based learning models for this group of mentors.

The convergence of formative and summative data suggests that as a whole, the time mentors invested in the training experience was worthwhile, supporting confidence, skill development, and meaningful connection among a group of workers from across the USA. Given that this training was designed to support a hard-to-reach and overburdened population, understanding the value of this training among mentor participants is significant to inform future tradeswomen supportive interventions.

Beyond immediate skill development, mentorship can have broader supportive impacts. In the case of this program, we heard from mentors that participation in the program boosted confidence and played a role in their decision to take on leadership roles within their locals and international union, contributing to greater representation of tradeswomen in positions of power. Mentors also reported a greater desire to get involved in their women’s committees as a result of participating. In contexts outside of the construction trades, scholars have noted the potential for mentorship to influence sociocultural norms outside of interpersonal mentor-mentee relationships, leading to positive culture shift (Mentorship Primer, n.d.). If sustained, mentorship programs for tradeswomen may have the potential to support increased recruitment and retention of tradeswomen participants, supporting gender equity in the trades.

### Benefits of mentorship for mentors

Although the goal of the training was to equip mentors with skills to pass on to apprentice mentees after the training, we learned that the training itself was a valuable source of support for mentors. Despite having more experience, journey-level tradeswomen, like apprentices, may be subject to the same feelings of isolation at work, while continuing to experience gendered hazards on the job as less experienced tradeswomen do (Turner et al., 2021; Zhang et al., 2021). This highlights the need for supportive programming for all tradeswomen. The range of experience levels in our mentor cohort was notable: the cohort represented journey-level workers who were newly journeyed out, those who had been working 5–10 years in the trade, and even some who were 30-year veterans in the trade. Bringing together tradeswomen with varying experience levels allowed the training experience to harness a broad range of tradeswomen stories. Below, we discuss design choices and environmental conditions we believe are important to consider for training for other tradeswomen audiences.

### Online setting and schedule

Although COVID-19 made remote delivery a necessity, this modality also made it possible to engage mentors from across North America in multiple time zones, giving mentors the chance to engage with other tradeswomen they may never have had the chance to meet otherwise. Conversely, remote delivery made it more challenging to facilitate interactive skill practice, as important cues like body language and tone are blunted in an online setting. This may also have potentially limited the degree to which rapport was built when discussing sensitive topics such as harassment, aggression, and discrimination, making it more difficult for mentors to speak up and feel comfortable engaging in open dialogue. The skillfulness of our trained facilitator was critical in building trust and a sense of safety during online sessions, highlighting the importance of a facilitator who has, or can gain, the trust of the group to approach sensitive topics. Given tradeswomen’s demanding schedules, it was important to provide training online and during the evening so participants could tune in while doing at home after work. This made it more possible for more mentors to participate, as they could tune in while completing household duties, eating dinner, or providing support to other family members.

### Senior mentor

The presence of the senior mentor was critical to the success of the training program. Given that neither the study team members nor the facilitator had a background in the trades, the personal stories she shared throughout the training sessions became an important factor in lending credibility and authenticity to the training. Mentors could turn to her for confidential support throughout the program knowing she understood the day-to-day reality of their working lives. Her support enriched the training experience and provided mentors with a knowledgeable resource to turn to if they had a challenging situation arise with a mentee.

## Limitations

Limitations of this work must be noted. First, the sample size is relatively small, with 27 mentors responding to the summative post-training survey over a three-year period. This highlights the competing demands on participants’ time and the challenge of engaging tradeswomen for multi-session, group-based training. The relatively small sample size and RCT study design of the parent study limit the ability to generalize findings to other populations. However, given the lack of research on tradeswomen focused interventions and the hard-to-reach nature of this population, reaching 27 mentors is significant.

Second, social desirability bias may have impacted survey results as the post-training survey was not anonymous. Study team members administered surveys through e-mail and played an integral role in scheduling and facilitating the live online training sessions, frequently interacting directly with mentors to provide scheduling assistance and ensure that all mentors had the training materials. This may have created potential bias in the summative post-training survey, which was not anonymous so that the study team could follow up with any mentors who reported challenges with the online training, and to ensure all mentors who completed the survey received compensation for completing the training.

Third, healthy worker bias may have played a role in which mentors completed the training. Those who already experience more support at locals with more tradeswomen representation, support among their local leadership, and those who have developed personal coping strategies may have been more able to participate in the training than tradeswomen who had less support at the individual, interpersonal, and organizational levels.

Last, the COVID-19 pandemic had a direct impact on the delivery of the training, causing us to pivot to an all-online delivery. While the online delivery made the training more accessible to a broader cohort of mentors, the wide-reaching impacts of the pandemic likely also influenced who felt able to participate and to what degree; for some, the disruptions to daily life may have made participating in the training more possible, while for others it would have been too burdensome. Round 1 of the training, conducted in 2021, was conducted under greater pandemic-related restrictions than round 2, which was conducted in 2023. The difference in environmental context between rounds may also have impacted mentor engagement. Constraints imposed by the pandemic created unique conditions in which this training was received, limiting generalizability of these results to other contexts.

### Recommendations and directions for future tradeswomen-focused mentorship

For future practitioners, we would recommend implementing certain changes to improve the delivery and uptake of the program. It is important to note that our reflections are specific to the constraints of the academic study structure in which this program was developed and deployed; practitioners at the union and local level may not face the same time-based constraints that we did while operating within the structure of a grant-funded study. The following recommendations may have varying applicability to local settings.

First, we recommend *integrating an in-person element* into the live training sessions. Although the remote nature of the program was a strength, we believe it would be easier to foster conversations about challenging topics like harassment and aggression on jobsites in an in-person environment, allowing for greater conversation depth. The benefits of the discussion-based training model may have been enhanced with greater trust and comfort that can be built in an in-person environment. Second, we encourage future iterations of the program to *set consistent groups for live training sessions* to allow each group to build rapport over the course of the training sessions. Although we deliberately held multiple training session times for each live session to be able to accommodate as many participant schedules as possible, having slightly different groups each time made it challenging for participants to build rapport and be able to jump into discussion. Third, the inclusion of our senior mentor was an important step to gaining credibility and trust among mentor participants, especially as we, the study team members, did not have lived experience as tradeswomen. In any future program, we recommend *finding a trusted and established tradeswoman* in your area who is interested in supporting the goals of the program, helping spread the word, and who can offer confidential support to mentors if any challenging situations arise. Fourth, given that many mentors make time for this role on top of full work and personal lives, we recommend pursuing opportunities to *compensate and/or recognize mentors* for their efforts. This could look like providing a stipend to mentors or creating a local-level certification for those who complete the mentorship training or work for a certain amount of time as a mentor.

Future research should focus on disseminating this training to a larger and broader group of tradeswomen from SMART International and piloting similar mentorship models in other trades with low tradeswomen representation. Based on feedback from mentors, more group-based, peer support models should be investigated as possible program delivery adaptations to deliver the benefits of mentorship to more tradeswomen.

## Figures and Tables

**Figure 1.**
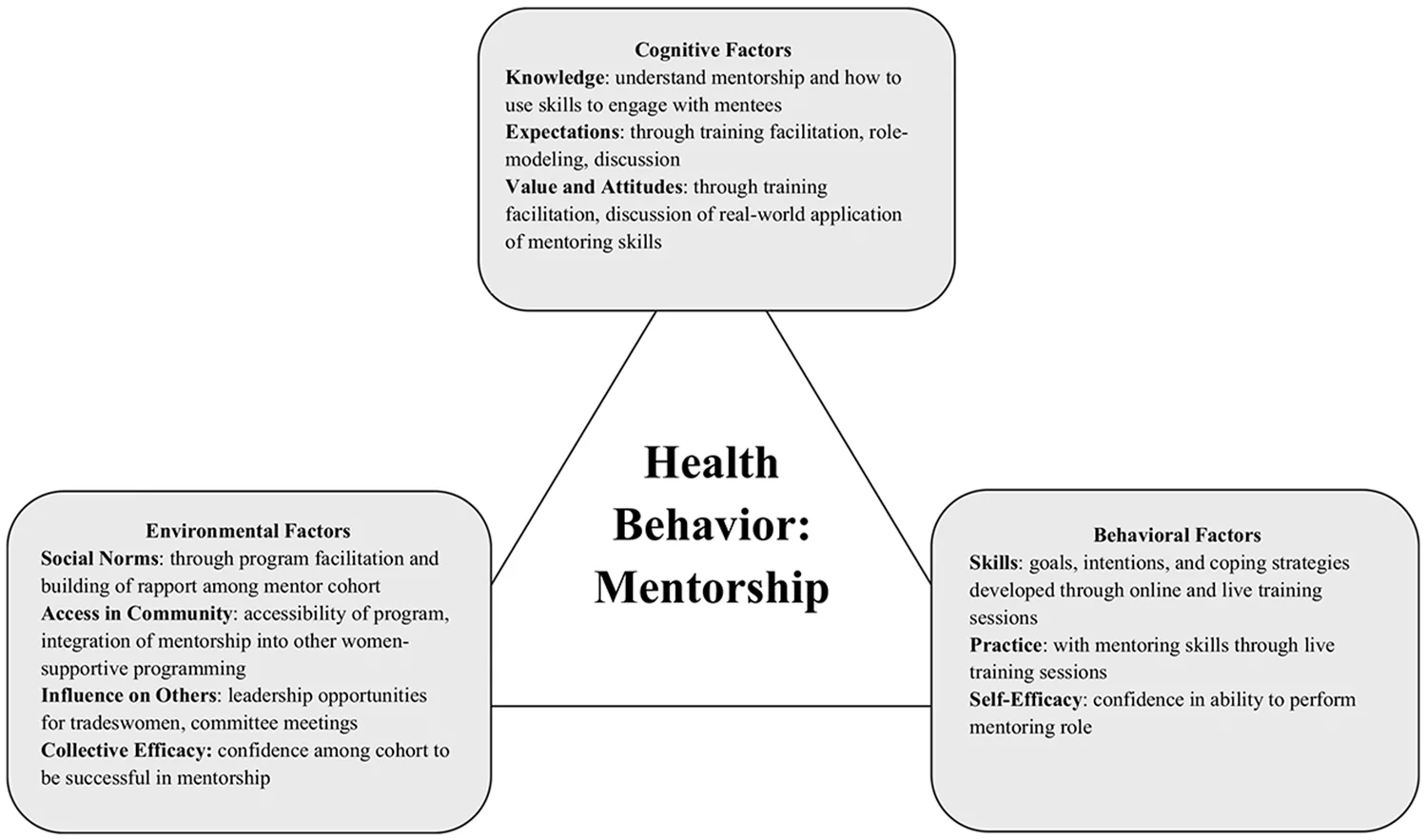
Applied social cognitive theory for mentoring.

**Figure 2.**
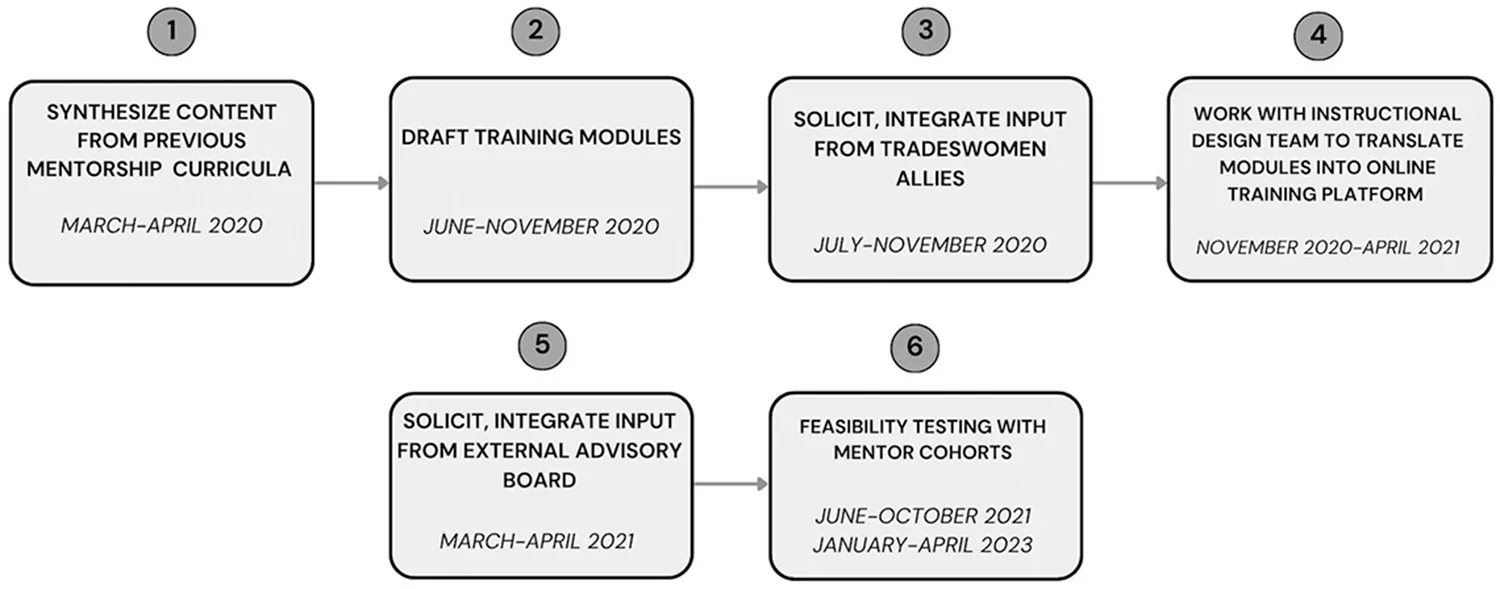
Training development process.

**Figure 3.**
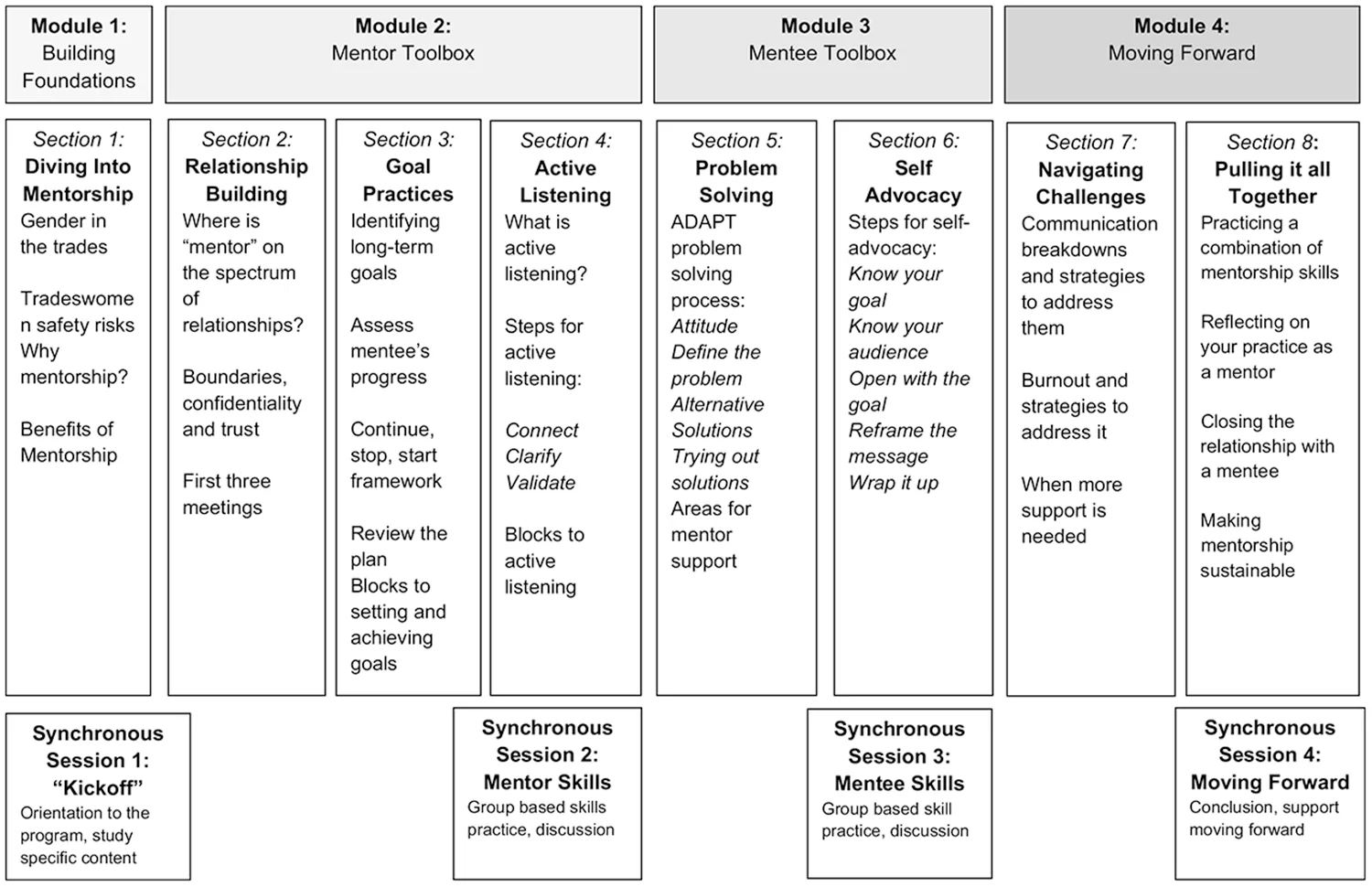
SMART mentorship training curriculum framework.

**Figure 4.**
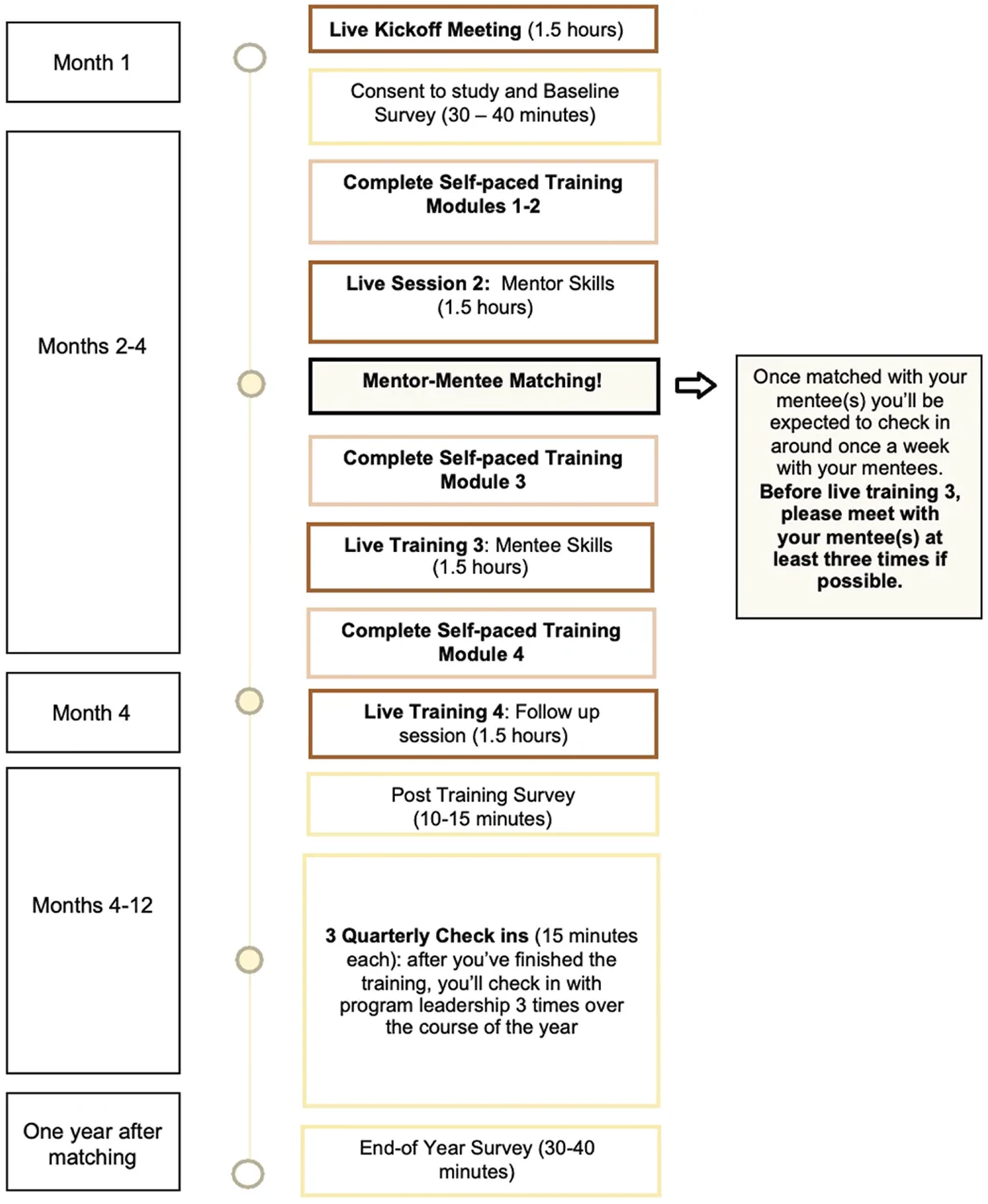
Timeline for mentor participation.

**Table 1.** Descriptive analysis of responses to multiple-choice questions in live training session and summative post-training evaluations

Evaluation measure	Total respondents		Survey question	Not at all n (%)	A little n (%)	Somewhat n (%)	Quite or very n (%)
Live Training Session 2	16	How valuable was today’s session?		0 (0)	1 (6)	0 (0)	15 (94)
Live Training Session 3	22	How valuable was today’s session?		1 (0)	1 (5)	2 (9)	19 (86)
Live Training Session 4	19	How valuable was today’s session?		0 (0)	0 (0)	0 (0)	19 (100)
Evaluation measure	Total respondents		Survey question	Not at all n (%)	A little n (%)	Somewhat n (%)	Very much n (%)
Summative post-training	27	How did the training change your confidence in the following skills?	Understanding the need for mentoring skills for women in the trades.	0 (0)	3 (11)	9 (33)	15 (56)
			Understanding the role of trust, boundaries, and confidentiality in a mentor/mentee relationship.	2 (7)	1 (4)	6 (22)	18 (67)
			Understanding the importance of goal setting for supporting mentee growth.	2 (7)	1 (4)	6 (22)	18 (67)
			Understanding how to use active listening skills to support my mentee.	1 (4)	2 (7)	10 (37)	14 (52)
			Understanding the process of problem solving (ADAPT) and feeling like I can use it to help my mentee.	0 (0)	1 (4)	12 (44)	14 (52)
			Understanding strategies I can use to help navigate challenges in mentorship.	1 (5)	2 (10)	9 (33)	15 (55)
			Understanding how to use a combination of mentoring skills.	0 (0)	1 (4)	9 (33)	17 (63)
		How often do you think you will use the following skills with your mentee?	Communication skills	0 (0)	0 (0)	4 (15)	23 (85)
			Goal setting skills	0 (0)	0 (0)	8 (30)	19 (70)
			Problem solving skills	0 (0)	0 (0)	3 (11)	24 (89)
			Self-advocacy skills	0 (0)	1 (5)	3 (15))	15 (75)
Evaluation measure	Total respondents		Survey question	Didn’t use ^1^ n (%)	A little n (%)	Somewhat n (%)	Very much n (%)
		How much did the following training components help you learn?	Self-paced online training modules	1 (4)	3 (11)	4 (15)	19 (70)
			Mentor Handbook	1 (4)	4 (15)	9 (33)	13 (48)
			Zoom training sessions	1 (4)	1 (4)	7 (26)	18 (67)

a Response options differed slightly between questions and evaluation measures.

**Table 2.** Content analysis of responses to open-ended questions in live training session and summative post-training evaluations

Evaluation measure	Open-ended question	Category of response	Illustrative Quotes
Live Training Session 2	*What could we do to make this session more worthwhile?*	Make sessions longer	“I really enjoyed the real-life example between [mentor] and [mentee], gave me a lot of insight on how a real-life situation could evolve and how to use the tools I have available. More examples and talking through the sections would always be beneficial.”
		More time for breakout rooms	
		More facilitation/discussion of real-world skill application	
Live Training Session 3	*What could we do to make this session more worthwhile?*	Provide more facilitation in breakout rooms	“Bring in someone to host breakout rooms. Conversation becomes pretty off topic. It is great for connecting and bonding with other mentors, although I feel the time could be more worthwhile if we stayed on topic.”
		Provide emailed handouts	
		More time	
		Provide solutions to mentee challenges	
Live Training Session 4	*What could we do to make this session more worthwhile?*	Send e-mail with list of mentor and mentee resources	“An email with a list of resources we can either use for ourselves and/or our mentee(s).”
Summative post-training	*What was the most valuable part of taking this training?*	Skill development/practice	“Breaking down the different issues, sorting them out, and constructively problem solving the problem. I liked how the active listening exercise was conducted to be used in a helpful and easy way to focus on what is said.”
		Discussion, connection with other mentors, study team	“Hearing other women share stories and obstacles [was valuable to me.] It was relatable to personal experiences and also offered new experiences that mentees might experience.”
		General training value	“Having a guide on how to be a mentor.”
	*Please provide any suggestions for how the training could be improved.*	Create more training opportunities	“Have an option for online training other than zoom, I wasn’t able to make it to as many sessions as I would’ve liked.”
		Expand engagement strategies	“Use the chat often. Give direction on what to share vs ‘who would like to share.’ Given the option, people like to sit back and just observe … ”
		Revise training content	“Handouts with distinct content for the different categories would be very useful to refer to later.”

## Data Availability

For access to the full online training and supporting documents, please visit https://phlearnlink.nwcphp.org/login/index.php. To enroll in the training, you must register for an account. Once registered, you will be able to access and view and work through the training, including a mentor handbook and best practices guide. Please contact the corresponding author at lilymm04@uw.edu with any questions about training access.

## References

[R1] Abid H, Arka G, Nateque MM, & Jamaluddin TM. (2021). Scientometric review of the twenty-first Century research on women in construction. Journal of Management in Engineering, 37(3), 04021004.

[R2] Agarwal PK. (2019). Retrieval practice & Bloom’s taxonomy: Do students need fact knowledge before higher order learning? Journal of Educational Psychology, 111(2), 189–209. 10.1037/edu0000282

[R3] Ariane Hegewisch EM. (2021). A future worth building: What tradeswomen say about the change they need in the construction industry. Institute for Women’s Policy Research. https://iwpr.org/wp-content/uploads/2022/02/A-Future-Worth-Building_What-Tradeswomen-Say_FINAL.pdf

[R4] Bandura A. (2023). Social cognitive theory: An agentic perspective on human nature. (Cervone D Ed.). Wiley. 10.1146/annurev.psych.52.1.1 11148297

[R5] Bartholomew Eldredge LK, Kok G, Markham CM, Ruiter RAC, Fernandez ME, & Parcel GS. (2016). Planning health promotion programs: An intervention mapping approach (4th ed.), [PDF]. John Wiley & Sons.

[R6] Bridges D, Bamberry L, Wulff E, & Krivokapic-Skoko B. (2022). “A trade of one’s own”: The role of social and cultural capital in the success of women in male-dominated occupations. Gender, Work, & Organization, 29(2), 371–387.

[R7] Bridges D, Wulff E, Bamberry L, Krivokapic-Skoko B, & Jenkins S. (2020). Negotiating gender in the male-dominated skilled trades: A systematic literature review. Construction Management and Economics, 38(10), 894–916. 10.1080/01446193.2020.1762906

[R8] Callaghan-Koru JA, & Aqil AR. (2022). Theory-informed course design: Applications of Bloom’s taxonomy in undergraduate public health courses. Pedagogy in Health Promotion, 8(1), 75–83. 10.1177/2373379920979684

[R9] CPWR Primary Training Materials. (2017, September 19). CPWR; The Center for Construction Research and Training. https://www.cpwr.com/research/training-and-awareness-programs-from-research/foundations-for-safety-leadership/foundations-for-safety-leadership-fsl-primary-training-materials/

[R10] CPWR Women in Construction. (2025, March 4). CPWR; The Center for Construction Research and Training. https://www.cpwr.com/research/data-center/the-construction-chart-book/interactive-7th/employment-income/women-in-construction/

[R11] Curtis HM, Meischke H, Simcox N, Laslett S, & Seixas N. (2016). P343 addressing health and safety risks for tradeswomen in the construction industry. https://oem.BMJ.com/content/73/Suppl_1/A236.3.short?casa_token=UYDgTkwloJcAAAAA:MD9S3FmsfJS3EO8_SKMIjnC2WLP4vQF617_-ugriVIkqPVWP0WRoHgu2ZFae69H_Rh42Hh37mTI

[R12] Curtis HM, Meischke H, Stover B, Simcox NJ, & Seixas NS. (2018). Gendered safety and health risks in the construction trades. Annals of Work Exposures and Health, 62(4), 404–415. 10.1093/annweh/wxy006 29471382

[R13] Curtis HM, Meischke HW, Simcox NJ, Laslett S, Monsey LM, Baker M, & Seixas NS. (2021). Working safely in the trades as women: A qualitative exploration and call for women-supportive interventions. Frontiers in Public Health, 9, 781572. 10.3389/fpubh.2021.781572 PMC8833840 35155345

[R14] Denissen AM, & Saguy AC. (2014). Gendered homophobia and the contradictions of workplace discrimination for women in the building trades. Gender & Society: Official Publication of Sociologists for Women in Society, 28(3), 381–403. 10.1177/0891243213510781

[R15] Employed persons by detailed occupation, sex, race, and Hispanic or Latino ethnicity. (2022, January 20). US Bureau of Labor Statistics. https://www.bls.gov/cps/aa2021/cpsaat11.htm

[R16] Employed persons by detailed occupation, sex, race, and Hispanic or Latino ethnicity. (2026, February 20). US Bureau of Labor Statistics. https://www.bls.gov/cps/cpsaat11.htm

[R17] Foley M, Oxenbridge S, Cooper R, & Baird M. (2022). I’ll never be one of the boys’: Gender harassment of women working as pilots and automotive tradespeople. Gender, Work, & Organization, 29(5), 1676–1691.

[R18] Glanz K, Rimer BK, & Viswanath K. (2015). Health behavior: Theory, research, and practice. (Glanz K, Rimer BK, & Viswanath K Eds.). (5th ed.) [PDF]. Jossey-Bass. https://ebookcentral.proquest.com/lib/washington/reader.action?docID=2082957&ppg=180

[R19] Goldenhar LM, Swanson NG, Hurrell JJ Jr., Ruder A, & Deddens J. (1998). Stressors and adverse outcomes for female construction workers. Journal of Occupational Health Psychology, 3 (1), 19–32. 10.1037/1076-8998.3.1.19 9552269

[R20] Goldenhar LM, & Sweeney MH. (1996). Tradeswomen’s perspectives on occupational health and safety: A qualitative investigation. American Journal of Industrial Medicine, 29(5), 516–520. 10.1002/(SICI)1097-0274(199605)29:5<516::AID-AJIM11>3.0.CO;2-3 8732926

[R21] Holdsworth S, Turner M, & Sandri O. (2023). Gender bias in the Australian construction industry: Women’s experience in trades and semi-skilled roles. Social Sciences (Basel, Switzerland), 12(11), 627. 10.3390/socsci12110627

[R22] Islam KF, Awal A, Mazumder H, Munni UR, Majumder K, Afroz K, Tabassum MN, & Hossain MM. (2023). Social cognitive theory-based health promotion in primary care practice: A scoping review. Heliyon, 9(4), e14889. 10.1016/j.heliyon.2023.e14889 PMC10070720 37025832

[R23] Kaskutas V, Dale AM, Lipscomb H, & Evanoff B. (2013). Fall prevention and safety communication training for foremen: Report of a pilot project designed to improve residential construction safety. Journal of Safety Research, 44, 111–118. 10.1016/j.jsr.2012.08.020 PMC3610407 23398712

[R24] Knowles M. (1984). Andragogy in action: Applying modern principles of adult learning. Jossey-Bass.

[R25] Krishna Nath Pandey SC. (2023). Andragogical interventions: Models for skill development. Zorba Books.

[R26] Mcdaniel R. (2010, June 10). Bloom’s taxonomy. Vanderbilt University. https://cft.vanderbilt.edu/guides-sub-pages/blooms-taxonomy/

[R27] Menches CL, & Abraham DM. (2007). Women in construction-tapping the untapped resource to meet future demands. Journal of Construction Engineering and Management, 133(9), 701–707. 10.1061/(ASCE)0733-9364,(2007)133:9(701)

[R28] Mentorship Primer. (n.d.). Google books. Retrieved June 11, 2025, from https://books.google.com/books/about/Mentorship_Primer.html?id=L_vSzAva8McC

[R29] Moir S, Thomson M, & Kelleher C. (2011). Unfinished business: Building equality for women in the construction trades. Labor Resource Center. https://scholarworks.umb.edu/lrc_pubs/5/

[R30] Ness K. (2012). Constructing masculinity in the building trades: “most jobs in the construction industry can be done by women”. Gender, Work, & Organization, 19(6), 654–676.

[R31] Niksadat N, Ghaffari M, Ramezankhani A, Rakhshanderou S, Farahani AV, & Negarandeh R. (2023). Experiences of patient education among people affected by cardiovascular disease: A qualitative study based on andragogy model. BMC Health Services Research, 23(1), 708. 10.1186/s12913-023-09622-1 PMC10308720 37386425

[R32] Porter J. (2019). Perceptions of and objective measures of gendered stress in construction [digital.lib.washington.edu]. https://digital.lib.washington.edu/researchworks/handle/1773/44804

[R33] PROGRAM - Mentorship Matters. (n.d.). Retrieved January 18, 2023, from https://mentorshipmatters.com/program/

[R34] Smith L. (2013). Trading in gender for women in trades: Embodying hegemonic masculinity, femininity and being a gender hotrod. Construction Management and Economics, 31(8), 861–873. 10.1080/01446193.2013.833339

[R35] Strecher VJ, DeVellis BM, Becker MH, & Rosenstock IM. (1986). The role of self-efficacy in achieving health behavior change. Health Education Quarterly, 13(1), 73–92. 10.1177/109019818601300108 3957687

[R36] Turner M, Holdsworth S, Scott-Young CM, & Sandri K. (2021). Resilience in a hostile workplace: The experience of women onsite in construction. Construction Management and Economics, 39(10), 839–852. 10.1080/01446193.2021.1981958

[R37] Vaismoradi M, Jones J, Turunen H, & Snelgrove S. (2016). Theme development in qualitative content analysis and thematic analysis. Journal of Nursing Education and Practice, 6(5), 100–108. 10.5430/jnep.v6n5p100

[R38] Wagner HE, & Kulwiec A. (2022). Expanding pre-apprenticeship training programs as a model to improve recruitment and retention of building construction tradeswomen. International Journal of Construction Education and Research, 18(1), 3–16. 10.1080/15578771.2020.1808547

[R39] Watts JH. (2009). “Allowed into a man’s world’ meanings of work-life balance: Perspectives of women civil engineers as “minority” workers in construction. Gender, Work, & Organization, 16 (1), 37–57.

[R40] Whitehead D. (2001). A social cognitive model for health education/health promotion practice. Journal of Advanced Nursing, 36(3), 417–425. 10.1046/j.1365-2648.2001.01973.x 11686756

[R41] Wright T. (2016). Support and solidarity: Networks and trade unions. In Gender and sexuality in male-dominated occupations (pp. 163–198). Palgrave Macmillan UK. https://download.e-bookshelf.de/download/0007/8709/94/L-G-0007870994-0014622041.pdf

[R42] Zhang RP, Holdsworth S, Turner M, & Andamon MM. (2021). Does gender really matter? A closer look at early career women in construction. Construction Management and Economics, 39 (8), 669–686. 10.1080/01446193.2021.1948087

